# Control of Pedicle Screw Placement with an Electrical Conductivity Measurement Device: Initial Evaluation in the Thoracic and Lumbar Spine

**DOI:** 10.1155/2016/4296294

**Published:** 2016-09-06

**Authors:** Olaf Suess, Markus Schomacher

**Affiliations:** ^1^DRK Kliniken Berlin Westend, Zentrum für Wirbelsäulenchirurgie und Neurotraumatologie, Berlin, Germany; ^2^Department of Neurosurgery, Charité University Hospital, Berlin, Germany; ^3^Vivantes Klinikum am Friedrichshain, Neurochirurgische Klinik, Berlin, Germany

## Abstract

*Aim*. Transpedicular screw fixation is widely used in spinal surgery. But the insertion of pedicle screws can sometimes be challenging because of the variability in pedicle size and the proximity of nerve roots.* Methods*. We detected intraoperatively the sensitivity for iatrogenic pedicel perforation with a hand-held electronic conductivity measurement device (ECD) that measures electrical conductivity of tissue-medium surrounding the instrument tip. ECD was used to guide the placement of 84 pedicle screws in 15 patients undergoing surgery for tumor or degenerative spinal disease at various spinal levels from T8 to L5. Additionally a CT-scan controlled screw positioning postoperatively.* Results*. The placement was “correct” (no mediocaudal pedicle wall penetration) for 78 of 84 (92,8%) screws, “suboptimal but acceptable” (0–2 mm penetration) for 4 of 84 (4,8%) screws, and “misplaced” (penetration > 2 mm) for 2 of 84 (2,4%) screws.* Conclusion*. Although this study was not designed to compare electronic conductivity technique to other guidance methods, such as fluoroscopy or navigation, a convincing “proof of concept” for ECD use in spinal instrumentation could be demonstrated. Advantages include easy handling without time-consuming setup and reduced X-ray exposure. However, further investigations are necessary to evaluate i.a. the economic aspects for this single-use developed instrument.

## 1. Introduction

**Figure 1:**
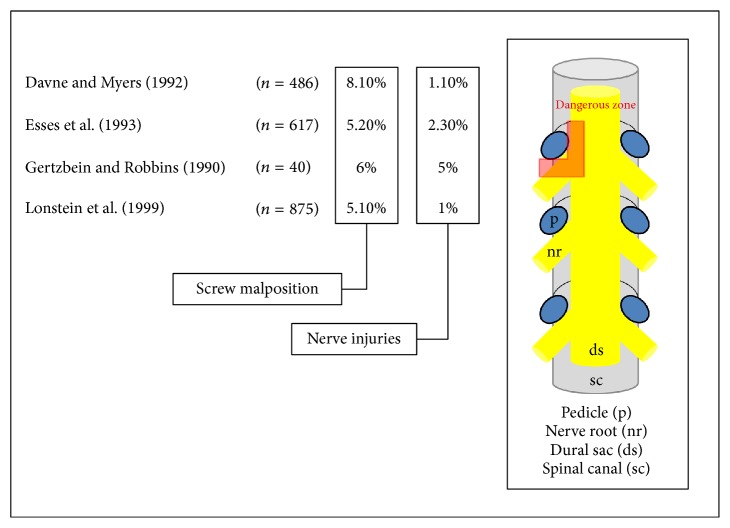
Illustration of the proximity of nerve root and dural sac to the pedicle. Misplacement of pedicle screws can cause damage to the dural sac or the exiting nerve roots if the pedicle wall is broken mediocaudally (dangerous zone). Modified figure published in PediGuard*™* booklet (SpineVision, Paris, France).

Transpedicular screw placement in the vertebra during posterior operations to stabilize the spine is currently the most widely used and successful technique to treat pathological changes of the spine caused by trauma, tumor, scoliosis, or degenerative diseases [1–5]. But correctly placing the pedicle screw in this operative technique can sometimes be challenging for the surgeon, due to variation in pedicle size and thickness at the various spinal levels and also due to the proximity to the nerve roots [6, 7]. Misplacement of the pedicle screws can cause damage to the dural sac or the exiting nerve roots if the pedicle wall is broken mediocaudally [8–11] (Figure 1).

Currently, various methods are used to attain the most correct pedicle screw placement possible. Among the mechanical aids, there is the possibility of probing the pedicle canal and inserting the pedicle screw by means of a guidewire [7, 12]. Among the imaging techniques, there is the controlled insertion of the pedicle screw during fluoroscopy or the use of computer tomography (CT) or other computer-supported navigation procedures [13–17]. But the use of intraoperative fluoroscopy can expose both the patient and the OR personnel to excessive radiation. Computer-supported navigation requires additional preoperative CT imaging, longer planning time, and more experience of the surgeon in the usage of navigation software and navigation-supported instrumentation [16]. Electrophysiological monitoring techniques, such as measuring motor evoked potentials (MEP) or electromyographic potentials (EMG), can be used to detect affections of the spinal nerves after insertion of the pedicle screw [18–21]. But sometimes, damage due to a misplaced pedicle screw can only be detected after dural or nerve root damage already occurred [22]. Also, they require additional qualified personnel [20].

Currently, an electrical impedance and conductivity measurement device was developed to improve the accuracy of pedicle screw placement. The probe-shaped tool can be used as a mechanical tool for the preparation of a lead canal for the insertion of the pedicle screw. It furthermore measures the electrical conductivity in the surrounding tissue, which changes depending on the tissue type, as shown in earlier animal and clinical feasibility studies [23–26]. These changes in conductivity are then communicated to the surgeon by light and sound signals, enabling the user to better understand the patient's spinal anatomy and to detect iatrogenic pedicle perforations prior to inserting the screw. In initial reports, this implement has demonstrated a high sensitivity (98%) and specificity (99%) for recognizing perforations of the cortex during pedicle screw placement [23–25]. On the other hand, it can be used for intraoperative electromyographic (EMG) monitoring through induction of a small electrical current on the surrounding tissue and nerve, during the localization and evaluation of spinal nerves and nerve roots.

This paper reports on our initial experience with this device in severe degenerative disease and spinal tumor surgery.

## 2. Methods

### 2.1. Device

**Figure 2:**
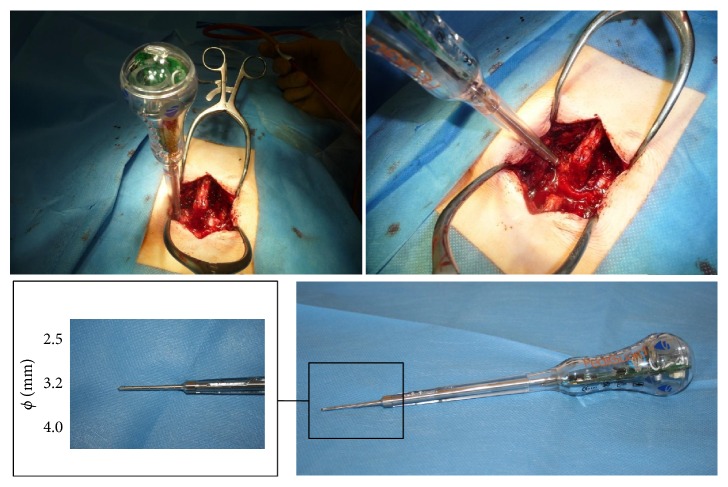
Pictures of intraoperative application in a case of lumbar spine surgery of the electrical conductivity device PediGuard (ECD) (SpineVision, Paris, France). The tip of the device comes in three sizes (diameter × length in mm): 2.5 × 40, 3.2 × 45, and 4.0 × 44.

**Figure 3:**
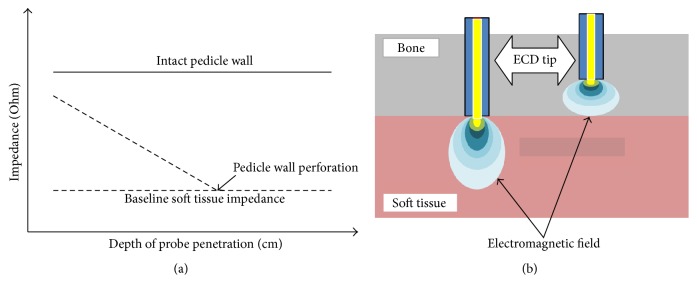
(a) Characteristics of the course of probe tip impedance values versus depth of pedicle penetration in an intact pedicle and one with pedicle wall perforation. The impedance values in the intact pedicle remain still above the soft tissue impedance while in perforated pedicle values drop down under the baseline of soft tissue impedance. Modified figure from published version in [22]. (b) Illustration of the electromagnetic field in two media with different electrical conductivity. In bone structure with low electrical conductivity the electromagnetic field is concentrated around the probe tip, whereas in soft tissue with higher electrical conductivity the electromagnetic field around the probe tip is spread out. Modified figure published in PediGuard booklet (SpineVision, Paris, France).

The electrical conductivity device PediGuard (ECD) (SpineVision, Paris, France) comes in three sizes (diameter × length in mm): 2.5 × 40, 3.2 × 45, and 4.0 × 44 (Figure 2), all of which can be used depending on the patient anatomy and level of segment. It is designed as a free-hand pedicle probe. The instrument tip serves as a bipolar electrode, which detects every 0.5 sec the changes of impedance/electrical conductivity in the surrounding tissue, due to changes of the electromagnetic field (Figure 3). These electromagnetic changes are transformed into audio and visual signals via an electronic switching circuit that is housed in the handle. For signaling there is both a speaker housed in the handle for indicating low or high frequency tones in various rhythms and also a two-color LED (green and yellow). A middle tone pitch and medium light frequency of the green LED are produced during the positioning of the instrument tip in the bone. During contact of the instrument tip with the cortex, a drop of the tone pitch and a decrease of the light frequency of the green LED occur, letting the surgeon know that the instrument tip is still in contact with bone tissue. If the cortex is broken through and the tip enters into surrounding soft tissue, a high pitch tone occurs and the green LED reaches a high light frequency, as a warning signal for the surgeon. Illumination of the yellow LED signals a malfunctioning in the measurement system.

### 2.2. Patients

The ECD was used to place a total of 84 polyaxial screws into 15 patients. The procedures were performed between October 2008 and October 2011. There were 6 male and 9 female patients with a mean age of 61 years (41–83 y). Patients with cardiac pacemakers and severe osteoporosis were excluded from participation according to the manufacturer's recommendations. In 8/15 patients a degenerative disease with DDD, spinal canal stenosis, and spondylolisthesis was the indication for dorsal instrumentation with a screw/rod system. In the other 7/15 cases a metastatic tumor of the spine (3x adenocarcinoma of the lung, 2x mamma carcinoma, 1x prostate carcinoma, and 1x hypopharynx carcinoma) was operated on. There were 7 single-level and 8 multilevel procedures (Table 1).

### 2.3. Surgical Procedure

**Figure 4:**
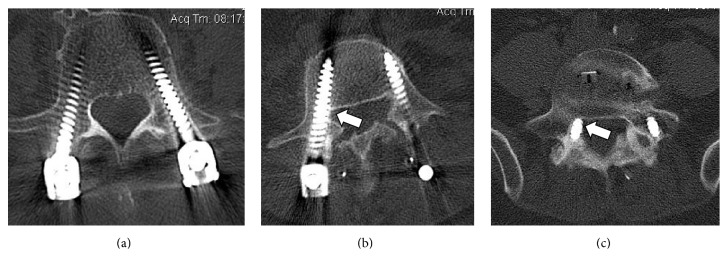
Examples of pedicle screw placement on postoperative CT imaging: (a) “correct” (no mediocaudal pedicle wall penetration), (b) “suboptimal but acceptable” (0–2 mm penetration), and (c) “misplaced” (penetration > 2 mm).

The instrument set was used for dorsal transpedicular stabilization operations on the thoracic and lumbar spine. The access to the vertebrae was in all cases via a dorsal midline access. The facet joints were prepared and an insertion canal was made with the ECD. Polyaxial screws (XIA, Stryker, USA, and Legacy, Medtronic, USA) with a diameter of 5.5 mm (thoracic) or 6.5 mm (lumbar) and a length of 30–50 mm were placed into the vertebral bodies according to the trajectory given by the ECD. Postoperatively, CT imaging of the spine (1 mm reconstructed slices, 0.7 mm increment, Kernel H70) was used to evaluate the positioning of the pedicle screw (Figure 4).

### 2.4. Data Evaluation

The placement of the pedicle screws on postoperative CT was evaluated by an independent radiologist and graded into three levels: (a) correct, (b) suboptimal (but acceptable), or (c) misplaced (Figure 4). The position of the pedicle screw was rated as “correct” when no screwthread penetration through the mediocaudal pedicle wall could be seen on the postoperative CT (1 mm slice thickness). The position was “suboptimal” (but acceptable) when the screwthread penetrated the pedicle wall less than 2 mm. The position was rated “misplaced” when the pedicle wall was penetrated 2 mm or more by the screwthread [7, 12].

## 3. Results

Thirty-eight screws (38/84; 45,2%) were placed during posterior instrumented fusion (PLIF) for spondylolisthesis, while the other 46/84 (54,8%) were placed during dorsal instrumentation for spinal tumor (Table 1).

The signal remained constant, while the ECD was advanced forward through the pedicle into the vertebral body in 72/84 (85.7%) pedicle sites. In the other 12/84 (14.3%) sites, sound and LED signals warned for variation in the measured conductivity as a sign for possible pedicle wall penetration. In these cases, the ECD was slightly moved backwards and redirected in another trajectory through the pedicle until no further warning signal occurred.

The screw placement was graded as “correct” for 78 of 84 (92,8%) screws and “suboptimal” for 4 of 84 screws (4,8%). Hence, 97,6% of the screws were satisfactorily placed, whereas 2 of 84 (2,4%) screws had to be graded “misplaced” (Table 2). In levels T8–T12 5.5 mm polyaxial screws were placed. For these cases the ECD with the 3.2 mm tip was used. Three out of the 32 thoracic screws were suboptimally placed with medial wall penetration of 1 mm and another T9 screw showed 2.5 mm misplacement. In levels L1–L5 6.5 mm polyaxial screws were placed. For these cases the ECD with the 4.0 mm tip was used. Two out of 52 lumbar screws were either suboptimally placed (1x at L4, Figure 4(b)) or misplaced (1x at L5, Figure 4(c)). The “misplaced” screw had a 2.5 mm breakthrough at the medial L5 pedicle wall without direct contact to the transversing nerve root. There was no clinical sign of radiculopathy immediately after surgery or on the 6- and 12-month follow-up examinations. No revision surgery was necessary. There were no mechanical failures of the ECD itself during these 15 operations.

## 4. Discussion

The use of transpedicular screw systems during spinal surgery has become widespread [2, 4, 5, 27], yet the insertion of pedicle screws can sometimes be a challenge for the surgeon because of the variability of size, height, and position of the pedicle in the various pathologies of the spine and also because of the immediate vicinity to exiting nerves and vessels [4, 5, 28]. With the increasing use of this procedure, there is also a rise of the complications associated with transpedicular screw fixation [29]. The operating surgeon must be experienced, in order to avoid risks, lasting deficits, or even reoperation for the patient [8, 9, 30].

In this study, there was a high rate of correct placement of screws with the ECD (92,8%) and a low rate of misplaced screws (2,4%). This is consistent with previously published reports [23–25], as well as gray literature from conferences [26]. Although this is an entirely respectable rate of accuracy, it is only marginally better than the rates previously reported in level-one studies on other methods of pedicle screw placement. The definitive benchmark is a recent meta-analysis on over 37,000 pedicle screws from 130 different studies [31]. That meta-analysis reported that 91.3% of all pedicle screws were accurately placed, and a subgroup analysis of the navigation-assisted in vivo pedicle screws had an accuracy rate of 95.2%. Similarly, an even more recent but narrower meta-analysis on about 7500 screws reported an overall accurate insertion rate of 89.2% and CT-navigation-based accurate insertion rate of 90.8% [32]. Since these recent meta-analyses, a new study evaluated the placement of 150 pedicle screws at T1–T3 using 3D-image guidance; they reported rates of 93.3% correctly placed screws and 6.7% as breaching the pedicle wall by 0–2 mm [33]. A very recent study on 424 lumbosacral pedicle screws placed with conventional open technique and intraoperative fluoroscopy reported correct screw placement for 93.2% of screws, questionable cortical encroachment for 2.8%, penetration ≤ 2 mm for 0.6%, and penetration > 2 mm for 1.6%. They concluded that “the conventional technique [of pedicle screw placement] still remains a practical, safe, and effective surgical method for lumbosacral fixation” [34]. Given our small sample size and the possible margin of errors, it cannot be concluded that ECD leads to a higher rate of accurate screw placement than conventional techniques, and indeed navigation-based screw placement appears to be slightly more accurate than ECD.

The present study has several important limitations that must be kept in mind. First, the study has no control group using other methods (such as fluoroscopy, navigation, or pure free-hand placement) as a basis for direct comparison. So we cannot draw any conclusions regarding improvement over conventional techniques. Thus, this study can only be regarded as a proof of concept for the electronic conductivity technique in spinal instrumentation. Second, the sample size is too small to serve as a conclusive evaluation, even if it had been designed as a randomized double-blind comparative study. Regrettably, it was not possible for us to enlarge the sample further. Third, the data were all from a single site; the rate of accuracy may be somewhat better or worse in the hands of other surgeons. Clinically, our initial experience was positive enough to warrant further investigation in carefully monitored research settings. We reemphasize the thought that this system has not yet undergone sufficient scientific evaluation for adoption into routine clinical practice (to our knowledge, randomized controlled multicenter studies are said to be initiated by the manufacturer but yet not published). Such RCTs have to prove that the ECD technology really improves patient safety in direct comparison to other conventional methods of pedicle screw placement.

Finally, we must comment on the cost of this device and associated design factors. The device cannot be reused for more than one operation—the outer casing of the device is made of a plastic, which makes it impossible to sterilize the instrument for reuse. The sound and light signals of the device are driven by a built-in internal battery that only lasts about 24 hours before dying out. In order to initially activate the battery, a paper-like tab on the side of the device must be pulled off. Once this paper-like tab is removed, the battery is active and running. There is no way to pause the battery from discharging. If the paper tab is accidentally removed before any operation is scheduled, the battery will die out anyway and the device would be useless. Furthermore, there is no way to open the device and replace the battery. Opening it up would require permanently breaking the plastic outer shell of the device, thus rendering it unusable. Moreover, the plastic shell has small airholes, so the user can hear the beeping tones from the speaker that is inside the handle. But consequently, blood or other patient fluids potentially carrying viruses can get inside the device through these sound-holes, thus contaminating the device so it cannot be safely reused on a second patient while the battery is still running. In the current era of dwindling healthcare budgets and limited resources for single-use instruments (e.g., in the DRG reimbursement system) one has to critically take this fact into consideration when deciding for such an “extra” tool. Even if this device later demonstrates a clinical benefit for patient health and safety (and not merely an increase in surrogate radiographic endpoints), rigorous health economic analyses are needed to determine whether it also has sufficient cost-benefit advantages. Potential ethnography endpoints for cost saving studies could include i.a. anesthetic case time and/or improved efficiency through lower instrument passes, leading to reduced overall operating time.

## 5. Conclusions

The general engineering concept of measuring electrical conductivity to improve the accuracy of pedicle screw placement appears, in our small initial clinical experience, to be a potentially useful method. The ECD safely allowed detection of changes in the electromagnetic field around the instrument tip as a warning signal for tissue with different consistency to bone. With careful handling, it even allows detection of cortical breaches before full penetration has occurred, giving the surgeon the chance to redirect the trajectory. Further advantages of this technique include easy handling without a time-consuming setup and no additional X-ray exposure. However, further studies should evaluate the advantages of the system in cost-comparison and clinical benefit, because in our viewpoint the economic inefficiency of a single-use product, which is otherwise quite promising, may limit the use in routine spinal surgery.

## Figures and Tables

**Table 1:** Indication for surgery, surgical procedure, number of screws, and segments operated on.

#	Indication	Procedure	Screws	Segments
1	Tumor (prostate)	Dorsal instrumentation	8	T8–L1
2	Degenerative	PLIF	4	L3-L4
3	Degenerative	PLIF	4	L4-L5
4	Degenerative	PLIF	6	L3–L5
5	Degenerative	PLIF	4	L4-L5
6	Degenerative	PLIF	4	L3-L4
7	Tumor (lung)	Dorsal instrumentation	4	T11–L1
8	Tumor (hypopharynx)	Dorsal instrumentation	8	T11–L4
9	Degenerative	TLIF	6	L2–L4
10	Tumor (lung)	Dorsal instrumentation	8	T8–T12
11	Degenerative	TLIF	4	L4-L5
12	Degenerative	PLIF	6	L3–L5
13	Tumor (mamma)	Dorsal instrumentation	8	T10–L2
14	Tumor (lung)	Dorsal instrumentation	6	T11–L2
15	Tumor (mamma)	Dorsal instrumentation	4	T8-T9

**Table 2:** Screw placements.

Level	Correct	Suboptimal	Misplaced	Total
T8	5	1		6
T9	4	1	1	6
T10	1	1		2
T11	10			10
T12	8			8
L1	6			6
L2	6			6
L3	12			12
L4	17	1		18
L5	9		1	10

*Total*	*78*	*4*	*2*	*84*

## List of Abbreviations

CT: Computed tomography
DDD: Degenerative disc disease
DRG: Diagnosis related groups
ECD: Electronic conductivity device
EMG: Electromyography
LED: Light-emitting diode
*L*(*x*): Lumbar spine level (*x*)
MEP: Motor evoked potential
mm: Millimeters
MRI: Magnetic resonance imaging
PLIF: Posterior lumbar interbody fusion
RCT: Randomized controlled trial
sec: Seconds
*T*(*x*): Thoracic spine level (*x*).
